# Description of a telephone and Internet-based intervention to improve community responses to COVID-19 spread

**DOI:** 10.1186/s41043-022-00325-7

**Published:** 2022-09-26

**Authors:** Galmangoda Najith Duminda Guruge, Nadeeka Rathnayake, Kalpani Abhayasinghe

**Affiliations:** 1 Department of Health Promotion, Faculty of Applied Sciences, Rajarata University of Sri Lanka, Mihintale, Sri Lanka; 2 Foundation for Health Promotion, Dehiwala, Sri Lanka; 3 Institute for Research and Development in Health and Social Care, Battaramulla, Sri Lanka; 4 Department of Nursing and Midwifery, Faculty of Allied Health Sciences, General Sir John Kotelawala Defence University, Ratmalana, Sri Lanka

**Keywords:** COVID-19, Empowerment, Lay community, Telephone and online communication

## Abstract

**Background:**

This paper describes the process and results of a health promotion intervention to engage lay communities using telephone and online communication, to improve their current responses to the spread of COVID-19.

**Methods:**

An intervention was conducted from March to July 2020 in three districts of Sri Lanka. Seven ‘trigger’ stories were shared through telephone or online communication to stimulate brainstorming and to engage selected community members. Determinants were identified and prioritised through discussions, and potentially beneficial actions were implemented as agreed by participants. The process was monitored, outcomes were evaluated monthly, and activities were modified according to ongoing observations.

**Results:**

A total of 638 families (both adults and children) involved actively in implementing useful actions and reported an increased sense of personal control. Potential risk groups, best feasible community safety precautions and preparation to face challenges in the event of infection reaching their community were identified during brainstorming sessions with community mobilisers. A majority reported that they felt more confident, united and less anxious about handling potential risks and problems. Other beneficial outcomes include lifestyle changes leading to healthier behaviours and a sense of greater control over the conditions that govern their lives.

**Conclusion:**

Use of telephone and online communication was effective in generating desirable community changes.

## Background

COVID-19, caused by the SARS-CoV-2 virus, has spread across the globe. National guidelines in Sri Lanka provide instructions for staff of health care and other sectors and the public regarding the management of its spread. Prevention of transmission is hindered by the rapidly spreading nature of the virus, delayed care seeking due to fear and stigmatisation and, most importantly, lack of awareness or interest among some members of the public in adhering to relevant guidelines. Generating a move to encourage the public to take greater control over preventing infection and dealing better with associated adverse consequences is therefore timely [1]. Lessons from the Ebola outbreak in Western Africa in 2014 show that community empowerment and raising awareness among the public had strong impact on controlling the spread and managing the outbreak [2–4].

A ‘health promotion’ (HP) approach allows community engagement and empowerment [1, 5–9] and promotes people taking control over the conditions that govern their lives to enhance well-being [1, 5, 7, 10]. The Ottawa Charter for health promotion (1986) [11] identified five action areas for health promotion: building healthy public policy, creating supportive environments, strengthening community action, developing personal skills and re-orienting health care services towards prevention of illness and promotion of health. Accordingly, the current study was carried out to evaluate the effectiveness of a health promotion intervention to engage lay communities using telephone and online communication, to improve their current responses to the spread of COVID-19. The intervention described in this study covers areas connected to community and personal aspects, and these can be shown to complement the policy and service delivery areas.

## Methods

### Approach and settings

A community-based HP intervention was carried out in three districts in Sri Lanka—Anuradhapura, Monaragala and Trincomalee. Four study settings (two communities from Anuradhapura and one each from the other two) were selected on the basis of previous exposure to HP activities and convenient access. The process was initiated during the nation-wide lockdown period and continued over five months (March–July 2020). Progress was monitored monthly using telephone and online communication platforms.

### Who were involved and what were their roles in the intervention?

The intervention team consisted of a group of university academics (*n* = 4) and community mobilisers (CM, *n* = 4) who had previously received training from the Department of Health Promotion, Rajarata University of Sri Lanka. The CM had collaborated in HP initiatives held prior to the advent of COVID-19 and had experience and skills in facilitating community activities gained over around eight years. The skills include the ability to start and maintain community processes, identify and address determinants, devise applicable indicators and measure progress throughout the activities. Academic researchers were mainly involved in conceptualising, creating ‘trigger’ stories, provide training and knowledge-based theoretical inputs to the CM and lay participants and documentation. The CM led the community-level intervention, carried out discussions with participants, monitored and evaluated the progress and facilitated communication between the academic research team and participants throughout the process.

### Trigger stories

The research team created a series of stories (*n* = 7) relating to the risks of COVID-19 transmission, safety precautions and useful preventive practices (See Table 1). Each story provided insights and triggers for the participants to generate ideas—for example, developing measures to assess the risk of transmission, working out actions that could be done at individual, family or community level and overcome possible challenges. Contents of the stories were developed using existing evidence and the World Health Organization (WHO) guidelines on COVID control.

**Table 1:** Summary of the contents of trigger stories

Episode	Content in brief
01	Introducing three families. In the first family, both mother and father work outside the home, the mother being a nurse; in the second family, only the father is employed outside—as a bus driver; in the third family all are farmers. Each family discusses likely ways of assessing the risk of getting COVID-19 infection based on their occupations, current understanding and level of exposures
02	Each family discusses the potential safety precautions that could be adopted to minimise the risk and to protect their loved ones if a family member became infected. For example, not sleeping in the same room or being physically close whenever the risk was considered high
03	Discussions among family members about the general guidelines (of the Ministry of health or WHO) regarding safety measures, sanitation, especially when one of the family members is working at a place where the risk of transmission is high
04	Neighbourhood families discussing how to care for and support each other. For example, helping economically disadvantaged families. The story suggests opening a charity shop in the village with essential food items or consumables so that more needy people can collect those free
05	Families plan to establish a community quarantine centre. This is a proactive measure to care for those who may be suspected of being COVID-19 carriers and have no facilities or space in their houses for proper self-isolation
06	The story is about people discussing the importance of taking measures to minimise the chances of elderly and those with non-communicable diseases being exposed to COVID-19—for example, youths taking action to protect their smoking friends and discourage smoking
07	In this story, the children start monitoring and recording the behaviour and adherence to safety measures by family members and neighbours. They assess whether their loved ones take safety precautions regularly

### Participant recruitment

Participant recruitment was carried out over the phone by CM, with a convenience sample. Those who had access to a telephone and/or online platforms such as WhatsApp, Viber, Messenger or SMS were invited to take part in the intervention. Verbal consent was obtained for voluntary participation. Telephone calls and online communication methods were used throughout the process. This was considered a pragmatic approach due to strict rules and regulations on people mixing with each other and the resultant difficulties of meeting people in person during the curfew and lockdown period. Using these methods to initiate HP interventions was a new experience for both the researchers and the participants.

### The process

**Fig. 1:**
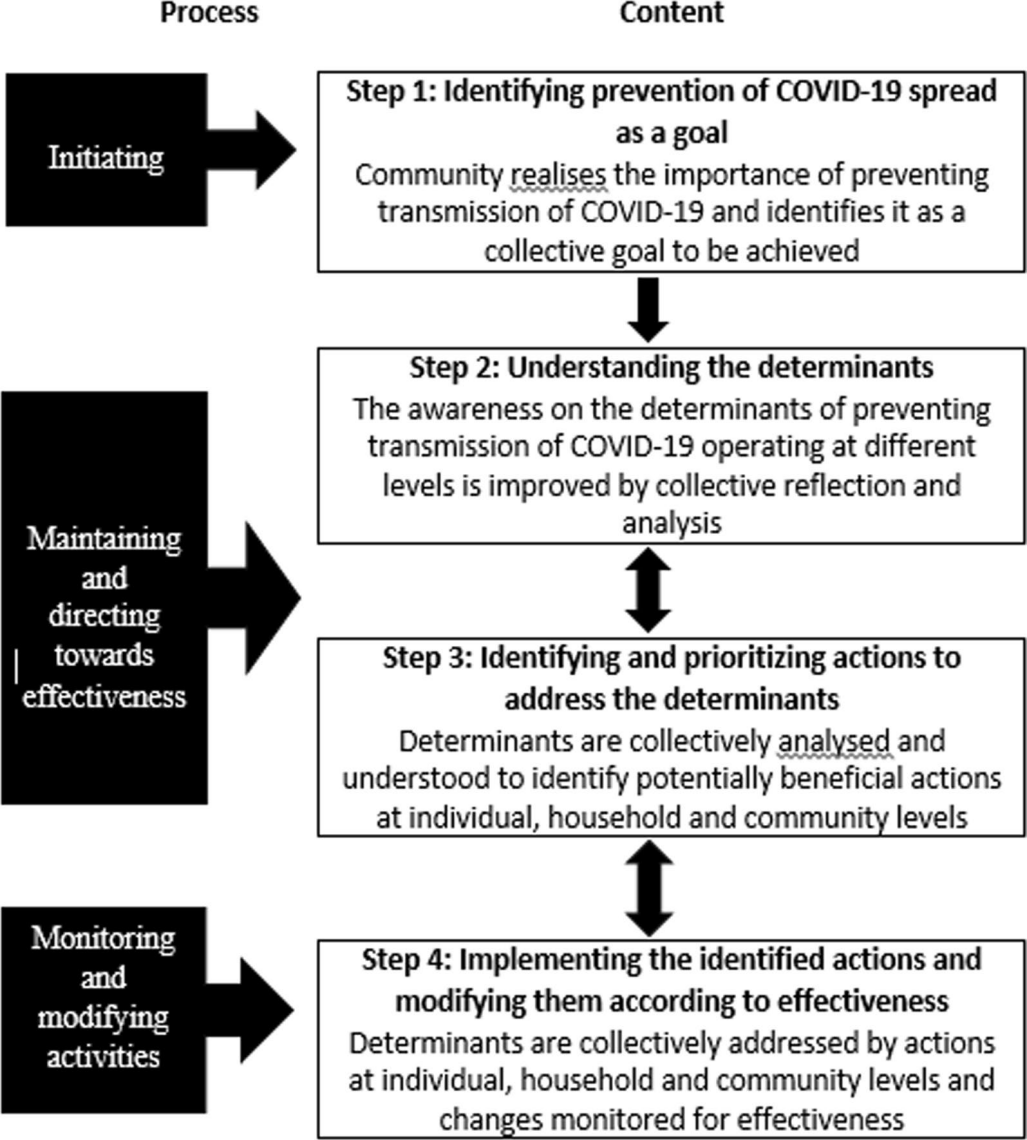
Conceptual framework for the intervention

Health promotion involves effective community actions in ‘setting priorities, making decisions, planning strategies and implementing them to achieve better health’ [11]. A community centred model of health promotion, introduced by Samarasinghe and colleagues in 2011 [12] and subsequently used in several studies [6, 7], was adapted for the present study. Figure 1 illustrates the conceptual framework utilised, which integrates components of the content (core subject matter) and the process (the flow of developments through CMs’ interaction with participants). Attention is paid to initiating, maintaining and directing a process towards effectiveness through continuous monitoring and ongoing modification of the process [6]. The key contents are described under four steps for clarity: deciding the goal, understanding the determinants, identifying and prioritising actions and implementing the actions.

#### Step 1: identifying prevention of COVID-19 spread as a goal

The ‘trigger’ stories were shared among the CM to initiate brainstorming by participants. During the first week, PI conducted several guided conversations over the phone with each CM (each call was approximately 20–30 min). PI explained to them the purpose, meanings and key messages given in each story. CM were encouraged to share how they would relate the scenarios to participants and discuss possible initiatives or actions that suited their context. After these brainstorming discussions, CM were asked to contact and engage participants. CM led the rest of the discussions with their community participants with minimal guidance of PI. It was expected that participants would realise the importance of preventing COVID-19 spread in their communities and a collective goal to strive for.

#### Step 2 and 3: understanding determinants, identifying and prioritising potentially beneficial actions to address determinants

CM carried out discussions with families in their setting and shared their views and knowledge. In later discussions (2–3 phone calls per week of approximately 30 min duration with each), they communicated other simple information too, such as how to perform hand washing, how to wear masks, what to do when sneezing and coughing, how to dispose the used masks safely, how to maintain physical distancing and how to prevent cross-contamination during day-to-day activities—as previously demonstrated by the academic team members. Added clarifications and feedback were given over the phone when required, with special attention to promoting collective actions [6]. It was assumed that the participants would identify and choose potentially beneficial actions on the basis of their priority needs, previous experience and capacity to implement them.

#### Step 4: implementing the identified actions and modifying them according to effectiveness

CM planned, and agreed with the participants, activities that could be implemented in their settings and steadily added knowledge as participants gradually implemented activities. Progress was monitored and the CM kept reflective diaries and took notes regularly and forwarded progress reports of the process to the PI every two weeks. PI and team provided feedback. Activities that needed alterations or changes were addressed and redirected accordingly. Intervention was continued for five months, and outcomes were evaluated continually and at the end.

All groups communicated through phone calls and online platforms throughout the process. There were hardly any physical gatherings. Financial support in the form of reimbursement of costs, such as for phone calls, was provided by the PI, when needed.

### Data collection and analysis

Data collection was carried out simultaneously over five months alongside the intervention. The main sources of data were reflective diary notes and progress reports submitted by the four CM. The diary notes included information about the progress, summaries of frequent conversations with participants and the notes of the CM.

At the completion of intervention, PI and team conducted nine in-depth, semi-structured interviews over the phone (approximately one hour each) with the four CM who led the interventions and with five randomly selected participants—to explore their experiences and observations throughout the process. Interviews were also used as a triangulation method to minimise information bias, as information received from CM and participants could be cross-checked. In addition, photographs of activities, the tools used to evaluate outcomes—for example, ‘Corona’ and ‘Well-being’ calendars (described in results section)—were also used as a secondary data source to facilitate the analysis [13]. Interviews were conducted until data saturation was judged to have been achieved [14]. All interviews were conducted in the mother tongue of the participants. Communications were audio recorded with verbal permission of the participants.

Audio recordings of interviews were transcribed verbatim. These transcripts and reflective diaries were analysed using thematic analysis. The academic team constantly compared the data and emerging themes from different settings for similarities and differences in each setting. Timelines of significant events during the intervention process were also considered when evaluating the progress of the entire approach [15]. Simple descriptive statistics were used to interpret findings obtained from the assessment tools.

## Results

### Process

The trigger stories generated interest and stimulated participants to think of actions that could be implemented in their households and community. At the end of the discussions, participants from all settings were able to recognise that preventing COVID-19 spread is important and they identified it as a worthwhile collective goal to achieve. Discussions facilitated the process of identifying, prioritising and analysing the determinants and planning intervention. These brainstorming sessions were also helpful in identifying potential challenges or difficulties and measures to minimise these, before implementing the actions within the community.

Three simple tools, namely the ‘Corona Calendar’, ‘Well-being Calendar’ and ‘Happiness Calendar’, were used for monitoring and evaluating the progress of the implemented actions. Participants developed the ‘Corona Calendar’ creatively using agreed criteria and colour codes. The ‘Well-being Calendar’ and ‘Happiness Calendar’ were tools previously used in other settings, for different HP interventions. Family members marked these calendars daily and monitored whether each individual adhered to those precautions or not and motivated those who do not follow the criteria to comply. For example, participants from Trincomalee used following criteria and the colour code for the ‘Corona Calendar’ to represent the risk of exposure:

- *Red if any family member went out unsafely. Orange for rest of the family members on that day.*
- *Pink for all the family members if any external person (a neighbour or a relative) visited the household.*
- *Green for all the family members if none of them went out and if no one visited the house. (CM 01—excerpts from diary notes).*

The ‘Corona Calendar’ is a tool to assess COVID-19 transmission risk in each household. This calendar assessed the risk of infection gaining entry into their house each time a family member or a visitor entered the house without adhering to safety precautions.

*Going out from home and having visitors were the two main risk factors. Corona calendar was an important tool, because, family members started monitoring the days they had to go out; they reduced going out and unnecessary visits. (CM 01)*

The ‘Well-being Calendar’ was used to assess the overall health and well-being of family members during the lockdown period. Participants assessed the family well-being in terms of level of adherence to healthy practices of each individual. For example, following behaviours or safety measures were assessed by the families in Trincomalee:

- *Washing hands frequently with soap or a disinfectant?*
- *Drinking boiled water?*
- *Covering face when coughing, sneezing and not spitting everywhere?*
- *Washing food items and other goods brought from outside?*
- *Following safety measures when leaving the home for work and also at work place?*
- *Disinfecting the watch, mobile phone, belt and shoes properly after coming home? (CM 01, excerpts from diary notes).*

People in Monaragala district used different criteria:

- *Following the safety precautions properly—Happy face.*
- *Following the safety precautions moderately—Normal face.*
- *Not following the safety precautions—Sad face.*
- *More than two family members go out frequently and not following safety precautions; high risk—Sad face (CM 02, excerpts from diary notes).*

The ‘Happiness calendar’ assessed the emotional well-being of each individual in terms of feeling happy, sad or angry, on a daily basis. People used colours and emojis of faces to represent the respective emotions:

- *Red colour—Angry face*.
- *Yellow colour—sad face*.
- *Green colour—happy face (CM 02, excerpts from diary notes)*.

CM were able to lead the intervention with minimal guidance of PI. Participants in all four settings engaged actively. The interest and engagement of families were higher in the other three communities when compared to Trincomalee, perhaps because those communities had previously been sensitised through previous HP interventions. CM were able to train other active community members without PI’s involvement and obtain their support to expand the group size and engagement. At the end of five months, a total of 638 families involved themselves actively in the process. Both adults and children from all settings collectively took part in the study. The majority were female. Two groups initiated the process from family level, while other two initiated it at the community level. Table 2 illustrates the participant engagement during the intervention. Tables 3 and 4 illustrate the progress of interventions in each setting.

**Table 2:** Participant engagement in the process from each setting

Setting	CM ID	Number of families engaged in the intervention					Reach
		March	April	May	June	July	
Trincomalee	CM-1	04	04	05	05	09	Family → Community
Monaragala	CM-2	05	32	44	67	129	Community → Family
Anuradhapura 1	CM-3	04	20	40	60	100	Community → Family
Anuradhapura 2	CM-4	05	15	150	300	400	Family → Community

**Table 3:** The progress of interventions in each setting

Activity	Trincomalee	Monaragala	Anuradhapura 1	Anuradhapura 2
Commencement	At family level from practicing safety precautions (They had no previous experience with HP interventions)	At community level from actions for smoking cessation and alcohol prevention	At community level from actions for smoking cessation	At family level from practicing safety precautions
Reach and tools developed and used for the intervention	Corona calendar Well-being calendar	Corona calendar Well-being calendar Happiness calendar	Corona calendar only	Corona calendar only
	*Corona calendar* Planning—1st month Implementation—2nd month onwards 2nd month—3 families 3rd–5th months—5 families *Well-being calendar* Planning—3rd Month Implementation—4th month onwards 4th month—5 families 5th month—5 families	*All calendars* Planning and implementation—3rd month onwards 3rd month—35 families 4th month—67 families 5th month—129 families	*Corona calendar* Planning and implementation—4th month onwards 5th month—15 families	*Corona calendar* Planning and implementation—3rd month onwards 3rd month—6 families

**Table 4:** Outcomes of the activities carried out over five months

Activity	Trincomalee	Monaragala	Anuradhapura 1	Anuradhapura 2
1st month	Understood that the virus can spread by exchanging food, money and other materials between households Started practicing safety precautions (Hand washing, over 1 m distance, wearing masks, prepared a separate place at home to wash hands) All families prepared and used a bowl for money transactions with vendors	People in 6 GN divisions designed and pasted posters in three village shops where cigarettes were sold (in 19 shops)	Took collective actions to prevent smoking and selling cigarettes *‘We discussed with three shops in the village. As a result, vendors stopped selling cigarettes. Some men bought cigarettes from two other shops at nearby villages. We talked to those two shops. They too stopped selling cigarettes’ (CM 03)*	Started practicing safety precautions *‘washed hands when returning home after going out,* *cleaned the tap after washing hands,* *placed their helmets and other goods outside/under bright sunlight’ (CM 04)*
2nd month	Implemented the corona calendar	Took actions to close alcohol bars	Identified a place to establish a ‘community quarantine centre’ with guidance and support from area MOH	Identified the risk of COVID-19 spread through exchanging money. Took action to prevent the risk
3rd month	Continued marking the corona calendar	Continued other HP activities they had initiated previously (e.g. NCD prevention, reducing alcohol consumption, improving child nutrition and happiness) during this lockdown period Families adhered to safety precautions	Started home gardening of vegetables and shared the harvest among neighbours *‘We hanged the vegetable packs at the fence so that neighbours could collect those.’ (P 03)*	2 families were under self-quarantine Discussed how to help them without spreading the infection
4th month	Implemented the well-being calendar	CM analysed the outcomes using simple statistical methods *‘67 Corona Calendars had been marked and according to analysis, 44 (65.7%) families were at lower risk, 20 (29.9%) families were at moderate risk and 03 (4.5%) families were at higher risk’. (CM 02, Diary notes)* Some started growing vegetables in their home gardens	Children involved in risk assessment; marking ‘Corona calendar’ and monitoring the adherence to safety precautions *‘Children observed and notified those who didn’t wear masks in public places. They complained when family members were going out without a mask.’ (CM 03)* Arranged a bowl of water and soap in front of houses for hand washing Village women sewed masks and distributed them among villagers free of charge	Actions for smoking cessation
5th month	Children in all families actively engaged in marking the ‘calendars’	Modified the ‘Corona Calendar’	Continued all activities initiated in previous months	Kept records of how much money they could save as a result of improved health and adherence to safety precautions (e.g. By limiting visits to shops and doctors)

### Outcomes

Outcomes can be described under two themes: (1) preventing infection gaining entry and (2) caring for and supporting vulnerable and economically disadvantaged families and families that may have to undergo quarantine.

#### Preventing infection gaining entry

All the community groups were able to identify the severity of the problem, behaviours and lifestyles that increased the COVID-19 transmission risk and ways to prevent spread. They realised the negative impacts of being infected with COVID-19 or being potential carriers.

*Arranged a discussion at MOH office with permission. Seven mothers from six GN divisions participated. We discussed about safety measures and importance of improving family wellbeing during this period. (CM 02)*

*Mothers were worried that many family members tend to go out when the curfew is lifted. They took measures to reduce it. (P 02)*

Perceptions on hindered day-to-day lives, obstacles to be faced such as quarantine procedures and possible death if infected with COVID-19 motivated all the groups to collectively create COVID-19 free homes and/or communities.

*Every family arranged a water tap or a water basin in front of their houses for hand washing (CM 01, Diary notes)*

*We limited visitors. Corona Calendars showed the increased risk on the days we had visitors. (P 01)*

The groups led by CM 01 and CM 04 identified that risk of transmission is high when purchasing goods from local vendors and they took measured to minimise that risk.

*We started using a bowl to exchange money without touching them when buying goods, especially the home deliveries. Some exposed the money to bright sunlight before putting them back in to their pockets and later disinfected the bowl. (P 01)*

*Risk of spreading COVID-19 was high when people gather at the drinking water filter. We kept a bowl there so that villagers could put money when buying drinking water. Displayed a notice with instructions, Put your money into the bowl. (CM 04, Diary notes)*

Participants in all community settings developed and adopted various forms of ‘Corona Calendars’, to assess COVID-19 transmission risk. Two groups developed ‘Well-being Calendars’ to assess their health and well-being. They modified the calendars from time to time, with more indicators.

#### Caring for and supporting each other

Three communities took collective actions to care for and support economically disadvantaged families and any families that may have to undergo quarantine.

*We grew vegetables and kept the extra harvest in front of our houses. We planted vegetables along road sides allowing those who needed to pluck them for free. (P 03)*

*Ten mothers collected food items [such as rice and lentils] from those who were willing to donate and distributed these among those who were under home quarantine. (CM 03)*

*We looked after the two quarantined families… Kept water bottles and groceries at their gates regularly. (CM 04, Diary notes)*

Some communities took measures to care for the elderly people with NCDs and took actions to protect vulnerable groups such as smokers or alcohol users. There was an active involvement and engagement of children in these activities.

*Elders and people with NCDs were at risk. We didn’t let them to go out unnecessarily. (P 05)*

Communities, other than in Trincomalee (CM 01), took action to stop selling cigarettes in their village shops. CM 02 and her group conducted poster campaigns to prevent smoking in their community.

*We pasted posters at public places… in front of shops. We received both positive and negative responses from villagers and shop owners. Towards the end of [1st month] posters were pasted in front of 19 shops in the area. (CM 02)*

As a result of these community actions, there was a reduction in smoking, alcohol consumption and incidents of family disputes or domestic violence.

*At the end of five months, twenty village men quit smoking, twenty men stopped using alcohol and over thirty were trying to reduce smoking and alcohol consumption. (CM 03, Diary Notes)*

*During this time government ordered to close alcohol bars. Most fathers did not take alcohol and therefore, mothers and children were happy. There was no quarreling. (P 03)*

*Fathers were at home, playing with children. They were happy. (CM 02)*

Some communities were able to identify at least one place that could be used as a basic community-led centre to care for infected individuals, those identified as potential carriers or recovered persons who are still subject to home quarantine.

*There is an abandoned house. Owners live [far away from village]. We spoke to them and got their permission to use that house as a [potential] quarantine centre. [CM 03]*

*We could find either a common place or a house that could be used as a quarantine centre. (P 04)*

### Impact

The impact can be described under two themes: (1) overcoming stigma, fear and undue anxiety regarding COVID-19 and (2) lifestyle changes leading to healthier behaviours.

#### Overcoming stigma, fear and undue anxiety

Findings indicate a reduction of stigma towards infected people, as well as COVID-19 suspects and those who were undergoing quarantine.

*Some families were suspected as COVID-19 carriers and asked to self-quarantine. They were cornered at the beginning. But later we started caring for them. I am not afraid of corona patients now as I know how to protect myself while caring for them as well. (P 04)*

People in all four communities reported that ‘*fear, stress and anxiety caused by COVID-19 was reduced*’, they ‘*felt more confident*’ and ‘*happier about handling potential problems and risks*’ when they were better informed and learned about the subject.

*I was very afraid at the beginning because I heard on the news that many people died in other countries. But now, I have much less anxiety, because I know well about safety measures to be taken. (P 01)*

*We now know well how to be protected when going out of home and when somebody arrives in our home. (CM 01)*

#### Lifestyle changes leading to healthier behaviours

Findings indicate many lifestyle changes leading to healthier behaviours and changing of thinking patterns and attitudes as a result of adherence to actions introduced during the intervention.

*Our children now wash their hands with soap and water before meals and after using the toilet, which they didn’t do earlier. (P 05)*

Other illnesses such as common cold, asthma and diarrhoea among children were reported less as a result of healthy habits such as frequent hand washing and wearing face masks.

*Now our children don’t get diarrhoea or flu as they used to get. Perhaps because they wash hands so often and wear masks. We could save lots of money spent for doctor visits and medication. (P 04)*

Some communities collectively took actions for smoking cessation, and reducing tobacco and alcohol consumption in the village in future also. People also considered about their psychological well-being and developed measures to assess their mental health during the lockdown period. As a result, child care, family happiness and well-being increased and incidents of family disputes and intimate partner violence were reduced as a whole. CM-2 and her group measured family happiness using a ‘Happiness Calendar’ and incidences that led to increased family happiness during the lockdown period:

*We started marking the happiness calendar. It was so useful to note everyday moods of our family members and family issues. (P 02)*

*Most village men stopped drinking alcohol, became less aggressive and quit beating their [wives and the children] (CM 01, Diary notes)*

*…family disputes were reduced as a result. (P 03)*

A majority reported that they felt *‘united’, ‘in more control’* and *‘had more power’* when working towards a common goal. Through this intervention, the participants could establish relationships built on trust, mutual sharing of knowledge, and bring together the wider community to achieve a common goal ensuring cultural sensitivity and sustainability. Table 5 shows the overall behaviour and attitude changes in each community as a result of our intervention.

**Table 5:** Overall behaviour and attitude changes in each community as a result of the intervention

Activity	Trincomalee	Monaragala	Anuradhapura 1	Anuradhapura 2
COVID-19 related	1. Reduced number of times they went out and visits to neighbourhood with the marking of corona calendar 2. Active engagement of children in assessing the COVID-19 risk 3. Fear and stigma towards COVID-19 infection reduced over time and people were united, started sharing, supporting and caring for others	1. Reduction of selling cigarettes in village shops 2. Active participation of families after seeing the improvement in other families the participants (e.g. reduction of cigarette and alcohol consumption, increased family unity and happiness) *‘Many families requested and joined the intervention when they got to know about it.’ (CM 02)*	1. Reduction of selling cigarettes in village shops (5 in total). Men reduced alcohol consumption or quit smoking 2. Attitude change—reduced stigma towards persons suspected to be infected with COVID-19 and increased compassionate care and support for those under quarantine	1. Reduction of smoking 2. Three village men reported they quit smoking at the end of the intervention, and said they will maintain this behaviour 3. Attitude change – *‘Tobacco use is stupid/foolish.’ (P 05)*
Non-COVID-19 related	1. Established new habits for day-to-day life (e.g. Frequent hand washing before meal and after using the toilet) 2. Reduction of respiratory tract infection and common cold due to good hygienic practices *‘Public Health Nursing Sister and some hospital staff said that there is a reduction of common cold and respiratory infections like flu among village children, perhaps due to good hygienic practices.’ (P 01)*	1. Reduced other diseases like influenza and diarrhoea as a result of adherence to safety precautions *‘Out of 129 families, 52 families (40.3%) reported that they have reduced other diseases like influenza and diarrhea’ (CM 02, Diary notes)* 2. Avoided buying unhealthy food items *‘We introduced the ‘healthy bag’ and ‘foolish bag’ concept when buying things. Many villagers started home gardening.’ (CM 02)*	1. Family happiness and unity increased	1. Reduction of respiratory tract infection and common cold due to good hygienic practices 2. Saving money by cutting down unnecessary purchases or visits to doctor 3. Family happiness and unity increased 4. Attitude change—Mothers started caring for their babies more than before, spent more time with them and paid attention to the illnesses of their children *‘We understood that children should be protected almost every day. Not only during this period.’ (P 05)*

## Discussion

Community involvement improves the way research is prioritised, commissioned, undertaken, communicated and used [4, 5, 16]. We intended to create a culture of active public involvement, where research is carried out jointly with or by members of the public rather than ‘about’ or ‘for’ them [6]. Community engagement is seen as critical in many health initiatives, but considered a fundamental component during disease outbreaks, communicable diseases and maternal and child health initiatives [17].

Applying a Health Promotion approach in crisis situations [1] will help to address COVID-19 spread at three different levels: downstream level—making individual behaviour changes; midstream level—interventions targeting communities and organisations, and upstream level—advocating for effective policies [18]. The present study mainly focused on downstream and midstream levels while upstream advocacy can now commence. Findings of the current study affirm that health promotion actions create living and working conditions that are ‘safe, stimulating, satisfying and enjoyable’ [11].

Findings of the current intervention indicated that lay people were empowered and enabled for better long-term handling of COVID-19-related issues, when they were better informed and involved in decisions and actions. The strategies used were effective and successful as people took the leadership and control over making positive changes to their lives. They demonstrated the ability to identify the causes and risk factors that increase COVID-19 spread and vulnerable groups who might get severely affected and the possible challenges they might face. They were able to take actions to minimise the risks of COVID-19 spread and find feasible, simple solutions to overcome the challenges with minimal guidance from the investigators. They showed greater resilience and were able to overcome their fears and worries during the lockdown period. People supported and cared for each other without discrimination and stigma. Their collective efforts and contributions made positive and healthy changes to lives of individuals, families and entire communities, improving reported overall happiness and general well-being of those who engaged in the process. Previous studies on community engagement approaches during epidemics such as Ebola, Zika and SARS support these findings and highlight the need for contextually appropriate community engagement strategies [17, 19].

Outcomes of the current intervention indicated that commencement and reach of interventions in the four communities were at different levels. Some communities involved in the current research prioritised actions to minimise the COVID-19 risk at home level, while others started with measures to prevent smoking and reduce alcohol consumption at community level.

The findings affirm that HP processes cannot be predetermined or carried out according to a prescription. It is a natural and organic process that enable people to grow [6, 12]. Findings indicated that the way people interact and live with each other, as well as their socio-cultural norms and beliefs require consideration when planning actions to respond effectively to COVID-19 outbreak as well. A recent study [20] reports that behaviour changes to reduce the community spread of COVID-19 are facilitated by incorporating them into existing routines, with guidance on how risk behaviours can be replaced by effective actions, rather than telling people how to avoid the risk.

This community-led intervention showed many positive impacts on people’s day-to-day lives. Participants were stimulated to start working for change and to incorporate new habits into their day-to-day lives, which was achieved through an ongoing, incremental process owned by them. This progress was achieved through ongoing monitoring, evaluation and feedback for continuing modification of activities. Evaluation was done using sensitive and innovative indicators such as the ‘Corona calendar’, ‘Well-being calendar’ and ‘Happiness calendar’.

The HP approach used in this intervention allowed the investigators to develop timely and community-focused research questions and objectives based on the experiences, needs and preferences of the participants involved. When compared to more researcher-led and researcher-oriented study designs, this process was led by the communities and their local mobilisers. Over a period of five months, we observed a high level of motivation and enthusiasm at individual, family and community level as everyone worked also towards monitoring and assessing results of their support to each other during the outbreak. Evidence from a systematic review by Brett et al., (2012) [16] shows that involvement of patients and public in most health and social care research helps to develop relevant research questions, user-friendly information and research tools, to determine more appropriate recruitment strategies for studies and also to interpret findings from the point of view of consumers.

### Strengths and limitations

A strength of this intervention was the involvement of CM as members of the steering group. They contributed as researchers and also as participants. Their role includes, for example, developing research questions based on their lived experiences, issues and challenges faced by them, developing widely accepted research materials such as tools for community intervention, identifying and recruiting participants, working with their communities throughout the intervention, monitoring progress over time and documenting preliminary findings.

Use of online platforms as the main communication method was effective as it saved time and expenses for travelling of the investigators, prevented the risk of transmission by physical gatherings and still kept people connected to each other during the nation-wide lockdown. Participants were capable of using smartphone and online applications such as Zoom or WhatsApp effectively and were not restricted due to inadequacy of IT literacy.

Restrictions on gathering as groups and face-to-face approaches for discussions and monitoring progress through direct observation created challenges. Innovative approaches such as the use of online platforms were required to overcome these barriers. It is claimed that online platforms may not provide interactions similar to face-to-face conversations as it requires certain digital skills [1, 21]. But we found that intervention using telephone and online platforms was effective in generating desired community changes. The researchers managed to interview participants over the phone, while some of the information relating to intervention tools and actions was obtained as images or photos.

## Conclusions and recommendations

The chosen HP approach to initiate a change in lay communities was successful as people took the leadership and control over positive changes in their lives. ‘Minimal contact’ engagement and involvement through telephone and online communication were effective in generating desirable community changes. The interest and engagement of families were at a higher level within communities which had previously been sensitised through other HP interventions. Both adults and children actively engaged in the process.

Participants took collective responsibility and implemented actions to prevent or minimise potential spread of COVID-19 in their households, neighbourhood and community. They were able to work out mutual safety measures to protect everyone in the village including vulnerable groups. They considered how to care for others and support any member of the community who may be suspected of being a COVID-19 carrier or those who were undergoing quarantine. They reduced discrimination and stigma directed towards COVID-19-infected people and felt more united and confident in control of dealing with COVID-19-related matters.

The intervention revealed that the public can take an active interest and implement useful actions in handling COVID-19-related issues. People can be empowered and enabled to deal with crisis situations with increased sense of personal control.

Based on the outcomes of the current intervention, we can recommend that training of active community members as community mobilisers is effective in promoting health and well-being in communities. Having trained health promoters available in the community will be an asset to control other communicable diseases, non-communicable diseases and in emergency or disaster responses. The authorities should consider investing in such training programs.

## Data Availability

The dataset used and analysed during the current study is available from the corresponding author on reasonable request.

## Abbreviations

CM: Community mobilisers
GN: Grama Niladhari
HP: Health promotion
NCD: Non-communicable diseases
MOH: Ministry of health
PI: Principal investigator
RUSL: Rajarata University of Sri Lanka
WHO: World Health Organisation
